# Effect of Brain Stimulation on Effort Is Task Dependent: Evidence From an HD‐tDCS Study on Cardiovascular Responses

**DOI:** 10.1111/psyp.70191

**Published:** 2025-11-24

**Authors:** David Framorando, Sarah Delobel, Andrea Razzetto

**Affiliations:** ^1^ Geneva Motivation Lab, FPSE, Section of Psychology University of Geneva Geneva Switzerland; ^2^ Swiss Center for Affective Sciences University of Geneva Geneva Switzerland

**Keywords:** approach/avoidance motivation, effort, high‐definition transcranial direct current stimulation (HD‐tDCS), pre‐ejection period

## Abstract

This study tested the effect of frontal hemispheric asymmetry (FHA) on mental effort in tasks of fixed and unfixed difficulty. To manipulate FHA, we applied high‐definition transcranial direct current stimulation (HD‐tDCS) to the dorsolateral prefrontal cortex (dlPFC) and assessed its impact on cardiovascular responses. Forty‐three participants performed two task conditions: (a) a fixed, easy task with a 50% accuracy criterion for success and (b) an unfixed, self‐paced task that delivered monetary rewards for each correct response. Before completing the tasks, participants received left cathodal versus right cathodal stimulation. Prior to each task, participants were told they could earn a moderate monetary incentive. We recorded cardiovascular responses, including pre‐ejection period (PEP), systolic blood pressure (SBP), heart rate (HR), and diastolic blood pressure (DBP). We expected right cathodal stimulation to induce left FHA. This should lead to higher effort when task difficulty is unfixed, due to increased participants' approach motivation and success importance. By contrast, no effect of stimulation was expected in the fixed and easy condition. As predicted, PEP reactivity—where more negative values indicate higher effort—was stronger in the unfixed task demand following right cathodal stimulation compared to the left cathodal stimulation and both stimulation conditions when the task was fixed and easy. These findings suggest that neuromodulation of the dlPFC alters effort intensity by shifting FHA, thereby increasing the perceived importance of success, which in turn determines higher effort under unfixed task demands.

## Impact Statement

This study enhances our understanding of approach motivation and effort by demonstrating that, in reward‐based tasks, task context modulates HD‐tDCS effects on effort‐related cardiac responses. Stimulation over the dlPFC increased pre‐ejection period reactivity (higher following right compared to left cathodal stimulation) within a self‐paced task context (each correct trial makes participants earn money). In contrast, when the reward could be earned by merely maintaining at least 50% accuracy, cardiovascular effort remained low after both stimulation conditions. These findings suggest that neuromodulation influences effort by modifying the importance of getting monetary rewards rather than via automatic sympathetic activation—a global autonomic response that would increase cardiac reactivity regardless of task demand.

## 1 Introduction

A large portion of our behavior is guided by rewards and punishments. For example, parents often promise their children ice cream for good behavior. However, sensitivity to rewards (SR) varies across individuals, with those exhibiting lower responsiveness frequently struggling to maintain motivation even in reward‐based tasks (e.g., Brinkmann and Franzen 2013; Franzen and Brinkmann 2015). Recent research has shown that SR can be enhanced through neuromodulation that induces greater left relative to right frontal hemispheric asymmetry (FHA; Harmon‐Jones and Gable 2018; Kelley et al. 2017), resulting in increased effort‐related cardiac responses during a task with monetary incentives (Framorando et al. 2025). Yet, transcranial direct current stimulation (tDCS) over the dorsolateral prefrontal cortex (dlPFC) may also produce automatic autonomic activation unrelated to motivational processes (Carnevali et al. 2020; Sesa‐Ashton et al. 2022). Accordingly, the present study aims to determine whether FHA‐induced changes in cardiovascular effort specifically reflect increased reward responsiveness rather than generalized sympathetic activation.

### 1.1 Motivational Intensity Theory

Motivational intensity theory (MIT), which is based on a resource conservation principle, suggests that effort invested in instrumental tasks for attaining personal goals depends on *task difficulty* and *success importance* (i.e., the importance of successfully performing the instrumental task) (Brehm and Self 1989). If task difficulty is clear and fixed, effort should increase with difficulty until the required effort exceeds what is justified by the importance of success. If effort exceeds the maximally justified amount, individuals should disengage to avoid wasting resources. If task difficulty is unclear or unfixed (i.e., the performer can choose any difficulty level), it cannot be used as an indicator of required effort. In these situations, individuals rely on success importance to minimize resource waste: the higher the success importance, the higher the effort. Success importance will not allow individuals to invest exactly what is required, but it will allow them to avoid investing more than justified (Richter 2013).

### 1.2 Rewards and Effort

Importantly, anticipated rewards can determine success importance, which in turn affects effort when success importance has a visible effect on effort—for example, when task demand is unclear or unfixed. For instance, in a task of unclear difficulty, participants who could earn higher monetary incentives exerted higher effort (e.g., Richter and Gendolla 2009). Notably, individuals with stronger SR should value more positive consequences of rewards. Consequently, the higher the SR, the stronger the effect of rewards on success importance. Research on anhedonia supports this idea (e.g., Brinkmann and Franzen 2013; Franzen and Brinkmann 2015). Dysphoric participants—who are characterized by low SR—exerted only low effort in tasks where participants can get a reward no matter the value of the reward. Other work replicated this effect with social rewards and in a group of participants suffering from major depressive disorder (e.g., Brinkmann et al. 2014; Franzen et al. 2019).

### 1.3 Approach Motivation, Sensitivity to Rewards and Effort

Of major importance, SR appears to be linked to the approach motivational system. Approach motivation encourages an organism to move toward an object (Harmon‐Jones et al. 2013; Lacey and Gable 2021). According to reinforcement sensitivity theory (Gray and McNaughton 2003; McNaughton 2024), approach motivation stems from the behavioral approach system (BAS), which responds to reward cues (Gray 1970). Heightened BAS activity is associated with increased SR. Importantly, neurobiological studies show that approach motivation involves greater left relative to right frontal hemispheric activity (FHA) as illustrated with EEG research that found that higher left frontal EEG asymmetry correlates with approach motivation measured with BIS/BAS questionnaires (Dawson et al. 1992; Harmon‐Jones and Allen 1997, 1998; Harmon‐Jones et al. 2009; Sutton and Davidson 1997). Importantly, noninvasive stimulation methods can shift these asymmetries to induce approach motivation (Fecteau et al. 2007; Ohmann et al. 2018; Knoch, Gianotti, et al. 2006; Kelley et al. 2017). TMS uses pulses to modulate brain activity contralaterally (Knoch, Gianotti, et al. 2006; Knoch, Pascual‐Leone, et al. 2006), while tDCS applies weak electrical current to alter excitability (Nitsche and Paulus 2000). More relevant, high‐definition tDCS (HD‐tDCS) uses a 4 × 1 ring configuration, isolating the stimulated region more precisely (Kuo et al. 2013; Parlikar et al. 2021). Configurations like cathode over right dlPFC surrounded by four anodes should increase left over right FHA (e.g., Framorando et al. 2025; Kelley et al. 2017; Parlikar et al. 2021).

Following this principle, initial evidence showed that brain stimulation can heighten SR and effort during reward‐based tasks (Framorando et al. 2025). Specifically, Framorando et al. (2025) used HD‐tDCS to increase approach motivation and recorded effort‐related cardiovascular responses. Guided by the neuromodulatory principle that right cathodal stimulation over the dlPFC decreases right frontal activity and increases relative left FHA, they predicted—and found in the female sample—that right cathodal stimulation would increase SR and effort compared with left cathodal and sham stimulation conditions.

Furthermore, recent studies have shown that neuromodulation may influence automatic sympathetic responses (Carnevali et al. 2020; Sesa‐Ashton et al. 2022). For instance, Carnevali et al. (2020) reported that anodal dlPFC stimulation—compared to sham—modulates heart rate and heart‐rate variability. Although heart rate indices are not pure measures of sympathetic activity, they are nevertheless influenced by it, raising the question of whether the stimulation of left or right dlPFC can directly affect sympathetic activation. These findings call into question whether the effects reported by Framorando et al. (2025) might be due to an impact of brain stimulation on autonomic responses, rather than a modulation of SR (Carnevali et al. 2020; Sesa‐Ashton et al. 2022). To examine this possibility, the present experiment tested the effect of left versus right cathodal stimulation in tasks of fixed and unfixed difficulty. This design controls for a potential global sympathetic activation produced by prefrontal neuromodulation (Carnevali et al. 2020; Sesa‐Ashton et al. 2022). According to MIT, neuromodulation should affect effort‐related cardiovascular indexes only when the task is unfixed. This is because in such a context, effort is directly determined by success importance, which is affected by SR. Conversely, stimulation should not influence effort in the fixed and easy condition. There, success importance should have no effect on effort.

### 1.4 Measuring Effort

Wright (1996) integrated MIT (Brehm and Self 1989) with the active coping approach (Obrist 1981). Effort intensity is reflected by beta‐adrenergic sympathetic nervous system impact on the heart. Beta‐adrenergic activity determines cardiac contractile force, measured as the cardiac pre‐ejection period (PEP)—the interval between ventricular depolarization and aortic valve opening (Berntson et al. 2004)—using impedance cardiography (ICG). Some studies have used systolic blood pressure (SBP) to measure effort, because cardiac contractile force affects cardiac output and the maximal vascular pressure following a heartbeat (Gendolla et al. 2012; Richter et al. 2016, for overviews). However, SBP is influenced by peripheral vascular resistance (Levick 2003). Other studies have relied on heart rate (HR) to monitor effort (e.g., Rogers 1969). However, HR is influenced by the parasympathetic nervous system. That is, PEP is the purest measure of effort among these indicators, because it directly reflects beta‐adrenergic sympathetic impact on the heart (Kelsey 2012), as illustrated by past work (e.g., Chatelain and Gendolla 2015; Framorando and Gendolla 2018, 2019a; Framorando et al. 2023, 2024a; Lasauskaite Schüpbach et al. 2014; Freydefont et al. 2012).

Importantly, PEP should be measured along with HR and DBP to monitor preload (ventricular filling) or afterload (arterial pressure) effects on PEP. PEP responses should be attributed to beta‐adrenergic sympathetic activity when decreases in PEP occur without simultaneous decreases in HR or blood pressure (Sherwood et al. 1990).

### 1.5 The Present Study

We investigated the effect of HD‐tDCS over dlPFC on effort‐related cardiovascular responses during mental concentration tasks of fixed and easy versus unfixed difficulty. Participants learned that they could earn money for their success in both tasks. Following the neuromodulatory principles observed with TMS, in which inhibition of the right hemisphere increases activity in the left hemisphere, left FHA was induced by applying cathodal HD‐tDCS over the right hemisphere (Right Cathodal Stimulation—RCS). On the contrary, right FHA was induced by applying cathodal HD‐tDCS over the left hemisphere (Left Cathodal Stimulation—LCS).

We hypothesized that RCS—which should determine higher left‐over‐right FHA—should increase approach motivation and heighten SR. Combined with moderate incentives, this should increase success importance leading to higher effort in a task of unfixed difficulty. In contrast, we anticipated that LCS—which should determine higher right over left FHA—in the same task would lead to moderate SR and success importance, resulting in lower effort. Finally, in a task of fixed and easy difficulty, success importance should not affect effort. According to MIT (Brehm and Self 1989), effort in fixed and easy tasks is primarily governed by low perceived task demand. As such, no effect of neuromodulation is expected.

## 2 Materials and Methods

### 2.1 Participants

To align with Framorando et al. (2025)—who demonstrated that 20 participants per cell were large enough to detect Bayes factors exceeding 10—we recruited 51 right‐handed female participants from various faculties at the University of Geneva.^1^ All participants completed the consent form and the BIS/BAS questionnaire. Of the 51 participants, only 43 showed up in the laboratory. In addition, following an initial check of signal quality and outliers, one participant was excluded from the main analysis due to cardiovascular data loss and one because of excessive PEP reactivity (+3 standard deviations above condition mean). This resulted in a final sample of *N* = 41 with a mean age of 21.72 years (SE = 0.34; median = 22.00; range = 18–25). Participants received CHF 30 (approximately 33.5 USD) as compensation for their time and participation.

### 2.2 Design

Participants were randomly assigned to one of the two stimulation conditions: left cathodal stimulation or right cathodal stimulation. Half of the participants first completed the fixed and easy task, followed by the unfixed task. The other half started with the unfixed task followed by the fixed and easy task. This setup created a mixed design, with stimulation condition (left vs. right cathodal stimulation) as a between‐subject factor and task (fixed and easy vs. unfixed) as a within‐subject factor.

### 2.3 Materials and Apparatus

#### 2.3.1 Physiological Measures

We used a Cardioscreen 1000 system (Medis, Ilmenau, Germany) to measure PEP and HR, both derived from synchronized ECG and ICG signals. Two pairs of electrodes (Ag/AgCl; Medis) were attached to the left side of the participants' neck and chest (left middle axillary line at the height of the xiphoid). The signals were amplified and digitized at a rate of 1000 Hz, then processed offline with a low‐pass filter set at 50 Hz using BlueBox 2. V1.22 software (Richter 2010). Automatic R‐peak detection was performed with a threshold‐based algorithm, followed by visual confirmation. The first derivative of the change in the thoracic impedance was calculated, and the resulting dZ/dt signal was averaged over a one‐minute period based on the detected R‐peaks. B point location was estimated on the basis of the RZ interval of valid cardiac cycles (Lozano et al. 2007), with all detections being visually inspected and manually corrected if necessary, following the protocols outlined by Sherwood et al. (1990). HR was determined based on the time intervals between heartbeats obtained with the Cardioscreen system.

SBP and DBP were measured at 1‐min intervals using a Dinamap ProCare monitor (GE Healthcare, Milwaukee, WI, USA). An automatic blood pressure cuff was placed on the non‐dominant arm, just above the elbow, and inflated automatically at 1‐min intervals.

#### 2.3.2 Application of HD‐tDCS

Neuroelectric StarStim 8‐channel device (Barcelona, Spain) was used as the main brain stimulation method. We used a 10–20 EEG system (Jasper 1958) to determine the locations of the target electrodes. To induce LCS, the cathode was placed over F3; the anodes over AF3, F1, F5, and FC3. To induce RCS, the cathode was placed over F4, whereas the anodes were placed over AF4, F2, F6, and FC4. HD‐tDCS was applied for 21 min at an intensity of 2 mA, including 30 s ramp‐on and 30 s ramp‐off for the current.

#### 2.3.3 Cognitive Tasks

The first task was an adapted version of a d2 attention task (Brickenkamp 1981). Participants indicated whether the stimulus was a “d” with exactly two apostrophes, which were displayed either above and/or below the target letter, by pressing “yes” and “no” response keys with two fingers of their dominant hand. Incorrect trials were composed of the letters “d” or “p” with 0, 1, 3 or 4 apostrophes or the letter “p” with two apostrophes. The second task was similar to the first one except that participants were asked to detect letters “b” with exactly two asterisks. Incorrect trials were composed of the letters “b” or “q” with 0, 1, 3 or 4 asterisks or the letter “q” with two asterisks.

#### 2.3.4 Tasks

##### 2.3.4.1 Fixed and Easy Task

Participants were informed that they could earn 7.5 Swiss Francs (9 USD) if their accuracy exceeded 50% of correct responses. Each trial began with a fixation cross displayed for 1000 ms, followed by the presentation of the letter for 3500 ms. If participants failed to respond within those 3500 ms, the feedback “Please answer faster” was shown for 3500 ms together with the updated success rate (the trial was recorded as incorrect). If they responded in time, they received feedback indicating either a correct or an incorrect trial—depending on the accuracy of their response—along with their current success rate. The trial ended with a blank screen lasting 2–5 s. The entire task consisted of 25 trials and lasted 5 min.

##### 2.3.4.2 Unfixed Task

Participants were informed that they could earn up to 7.5 Swiss Francs (9 USD) in total. Each trial started with a fixation cross displayed for 1000 ms, followed by the letter which remained onscreen until participants responded. They were told that fast and correct responses (< 484 ms) would yield 0.30 Swiss Francs (USD 0.36) per trial, slower but correct responses (> 484 ms) would yield 0.10 Swiss Francs (USD 0.12), and incorrect responses would yield 0.00 Swiss Francs. After each response, feedback appeared for 3500 ms, indicating whether the participant responded quickly and correctly, slowly and correctly, or incorrectly, along with their cumulative earnings. Each trial stopped with a blank screen lasting 2–5 s. The task ended after 5 min.

### 2.4 Procedure

The local ethics committee approved all the procedures and measurements before data collection started. The experimenter was hired and remained blind to both the hypotheses and experimental conditions to avoid experimenter effects (e.g., Gilder and Heerey 2018). Before starting the experimental sessions, participants responded to a safety questionnaire to report whether they had a pacemaker, any medical implants, or a history of brain damage. Also, they were required to read and sign a written informed consent form outlining the potential effects of tDCS and detailing the experimental procedures. In addition, they were asked to complete the BIS/BAS scale (Carver and White 1994). For safety reasons, individuals with epilepsy, headaches, or metallic implants in the head were excluded.

On arrival, participants were welcomed by the experimenter and were asked to sit in a comfortable chair in front of a computer. The experimenter then started the computer program with the experimental protocol (Nuxt.js; Version 3.12.4, NuxtLabs, San Francisco, CA, USA). Before starting the experiment, the participants were equipped with the tDCS and physiological sensors.

**FIGURE 1:**
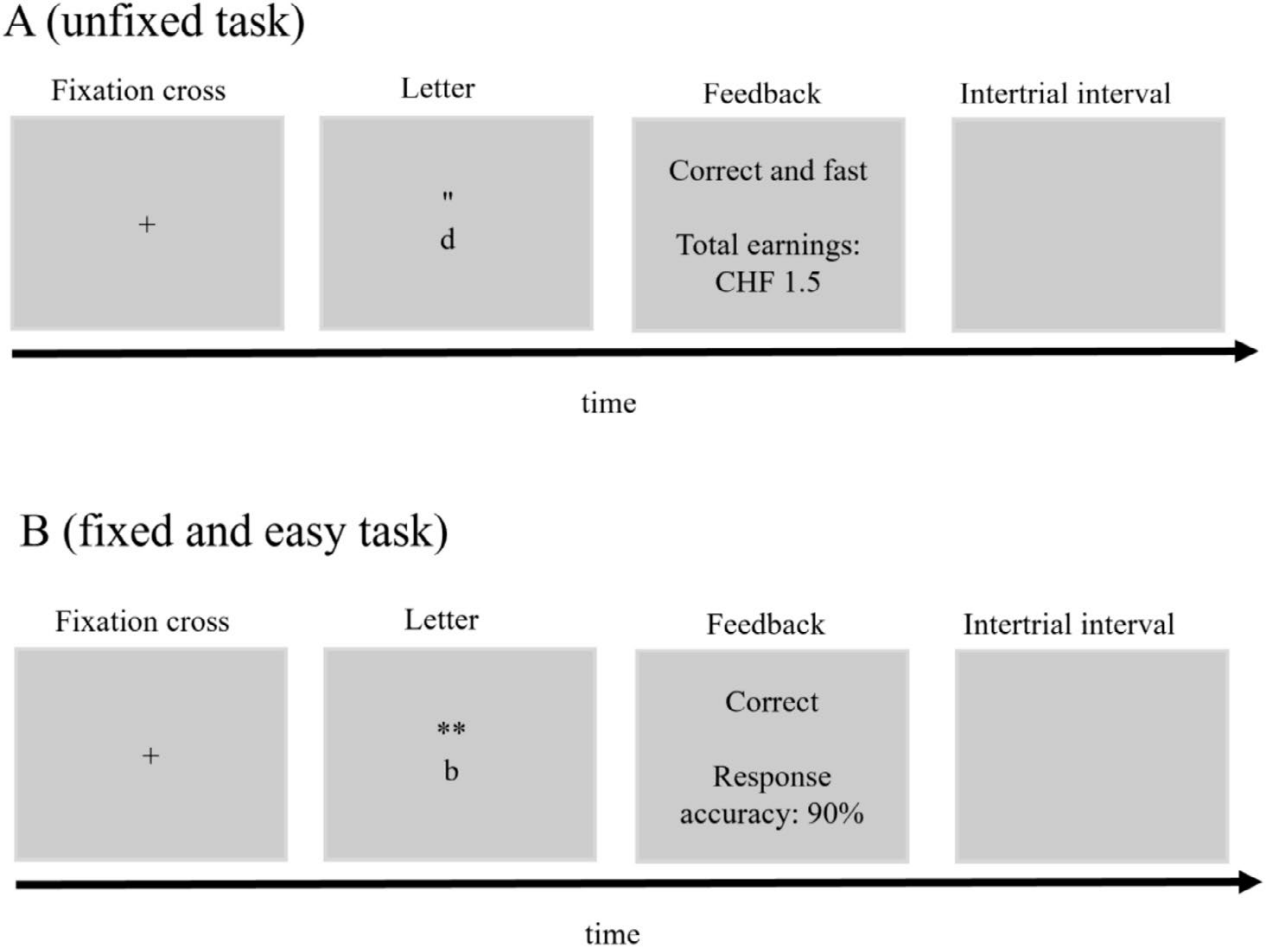
Example of an experimental trial. In the example, (A) corresponds to task 1 in the unfixed condition, while (B) corresponds to task 2 in the fixed and easy condition. In (A), the letter d is accompanied by exactly two apostrophes. Participants should respond “yes” by pressing the green keyboard key. Similarly, in (B), the letter b is accompanied by exactly two asterisks. Participants should respond “yes” by pressing the green keyboard key.

Participants were first asked to report their age. They then watched a hedonically neutral documentary about space (20 min) while tDCS was administered. This was followed by another neutral documentary about trees (7 min), during which cardiovascular baseline activity was recorded. Participants were instructed to remain passive and relaxed during this period. After the baseline period, participants completed the first version of the d2 attention task, while physiological measures were recorded. This was followed by a second baseline period, during which participants watched a short documentary about lemurs (3 min). No physiological data were collected during this phase, which served to reset physiological activity to baseline levels. Following this, participants performed a second version of the d2 attention task (see Section 2.3.4). The experimental protocol is depicted in Figure 1. After completing the second attention task, participants answered additional questions about their native language, French language proficiency and medication use. The experiment concluded with a debriefing session, during which participants were informed about their scores, whether they succeeded in the task, and were also asked to guess the purpose of the study. They were also questioned about any pain experienced during the HD‐tDCS procedure.

### 2.5 Data Analysis

The data and data coding for the reported study are available on the Geneva Motivation Lab server: https://amb5.genevamotivationlab.ch. The data analysis process was preregistered on the Aspredicted platform (https://aspredicted.org/hwbd‐67x4.pdf). Analyses were performed using RStudio (R Core Team 2023) and JASP (JASP Team 2023) software.

To examine the effects of HD‐tDCS on cardiovascular responses, we used Bayes factors to compare our hypothesized effects against the null hypothesis (Masson 2011). This Bayesian approach was chosen both to maintain consistency with prior analyses used in Framorando et al. (2025) and because it allows for the quantification of evidence supporting either the null or alternative hypothesis, offering a more nuanced interpretation of the data than traditional null‐hypothesis significance testing (Dienes 2014; Rouder et al. 2009). We hypothesized that right cathodal stimulation (RCS) would lead to higher effort‐related cardiovascular reactivity compared to left cathodal stimulation (LCS) in the unfixed task, as well as compared to both stimulation conditions in the fixed and easy task. To test this, we applied an a priori contrast with weights of −3 for the RCS condition in the unfixed task, and +1 for LCS in the same task condition and for both stimulation conditions in the fixed and easy task.

## 3 Results

### 3.1 BIS/BAS Scores

To check for potential dispositional differences in approach/avoidance motivation, preliminary one‐way Bayesian ANOVAs were conducted with HD‐tDCS condition as the factor for BIS, reward responsiveness, drive, and fun‐seeking scores. The resulting Bayes factors and posterior probabilities were low for BIS, reward responsiveness, drive, and fun‐seeking scores (BFs ≤ 0.852; *p*(*M*∣data) < 0.460). These findings show low support for condition‐based differences in BIS/BAS scores at baseline.

### 3.2 Cardiovascular Baseline

We had a priori decided to calculate participants' cardiovascular baseline values by averaging the measures taken during the last 3 min of the habituation phase (see Framorando et al. 2023, 2024a, 2024b), which showed high internal consistency (Cronbach's *α* ≥ 0.95). This period was chosen because cardiovascular activity generally stabilizes toward the end of the habituation period. To examine any baseline differences across HD‐tDCS conditions, we performed one‐way Bayesian ANOVAs on the baseline scores for each cardiovascular index. The analyses yielded low Bayes factors and posterior probabilities (BFs < 0.473; *p*(*M*∣data) < 0.321), indicating minimal evidence for condition‐based differences at baseline. Mean and standard error values for each cardiovascular index at baseline are presented in Table 1 for each stimulation condition.

**TABLE 1:** Cell means and standard errors (in parentheses) of the cardiovascular baseline scores.

	LCS	RCS
	*M* (SE)	*M* (SE)
PEP (in ms)	100.32 (1.84)	102.95 (2.20)
SBP (mmHg)	100.70 (1.74)	101.57 (1.77)
HR (beats/min)	73.81 (1.57)	76.42 (1.94)
DBP (mmHg)	61.94 (1.26)	62.12 (1.38)

*Note:* *N* = 41 for all cardiovascular indicators.

Abbreviations: DBP = diastolic blood pressure, HR = heart rate, LCS = left cathodal stimulation, PEP = pre‐ejection period, RCS = right cathodal stimulation, SBP = systolic blood pressure.

### 3.3 Cardiovascular Reactivity

We calculated cardiovascular reactivity scores by subtracting the participants' baseline scores from the average values of the five 1‐min measurements of PEP, SBP, HR, and DBP assessed during task performance, which showed high internal consistency (Cronbach's *α* ≥ 0.96). We conducted 5 (time: task minutes) × 2 (stimulation: left cathodal, right cathodal) × 2 (task: fixed and easy, unfixed) mixed‐model Bayesian ANOVAs for each physiological measure to detect potential interactions between time, stimulation and task. For all cardiovascular parameters, model testing the time × stimulation, time × task, time × stimulation × task interactions yielded low Bayes Factors and posterior probabilities (BFs < 0.273, *p*(*M*|data) < 0.082). In addition, we included order (whether participants started with the fixed and easy task or the unfixed task) as a covariate. Also, testing models with order, order × stimulation, or order × stimulation × task resulted in models with low Bayes factors and posterior probabilities (BFs < 0.653, *p*(*M*|data) < 0.119). Consequently, subsequent analyses were conducted using the mean cardiovascular reactivity across the five 1‐min intervals for PEP, SBP, HR and DBP, without including task order as a factor.

In the unfixed task, there was no predetermined number of motor responses. Therefore, response frequency varied across participants, raising the question of whether this variability might explain potential differences in cardiovascular reactivity. Accordingly, we ran Bayesian ANCOVAs for each cardiovascular index including the number of motor responses as a covariate. This yielded low Bayes factors (BFs < 0.788) and low posterior model probability (*p*(*M*|data) < 0.202), suggesting that variation in motor responses is unlikely to account for variability in cardiovascular reactivity patterns.

#### 3.3.1 PEP Reactivity

**FIGURE 2:**
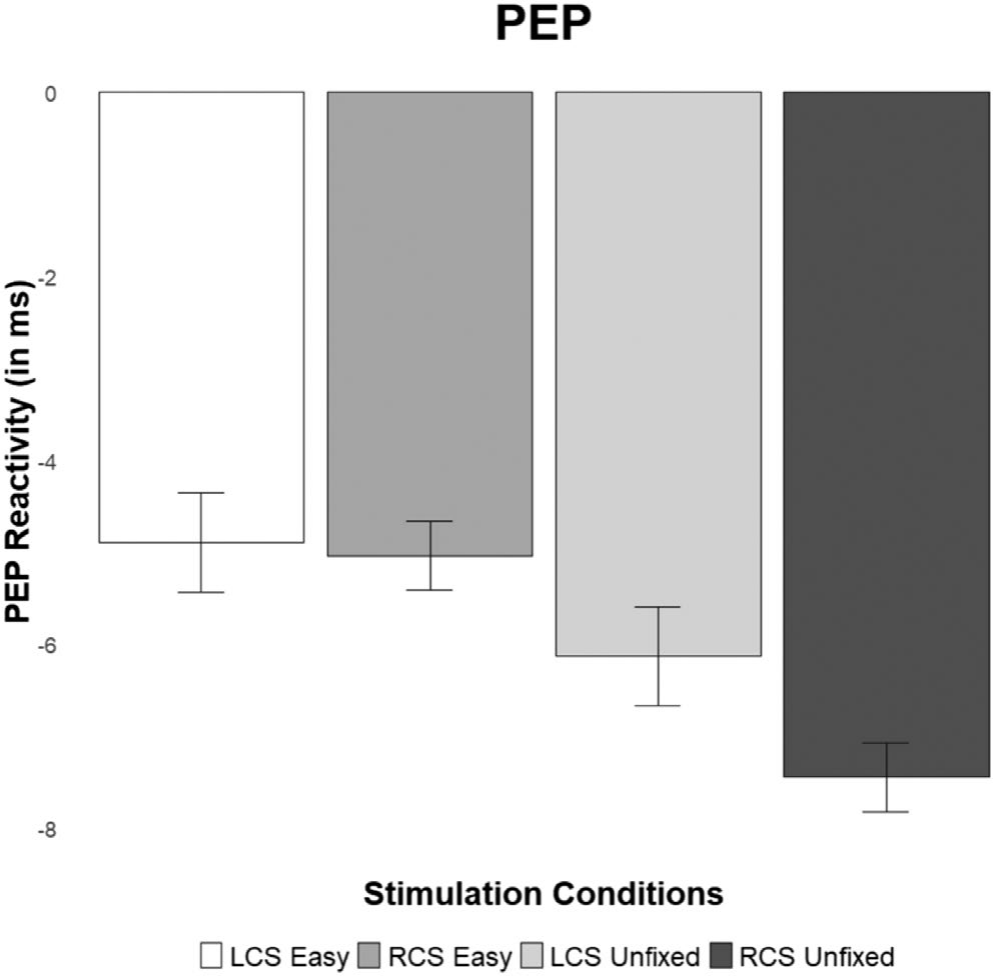
Cell means and ±1 standard errors of pre‐ejection period (PEP) (in ms) reactivity in the experimental conditions (LCS = left cathodal stimulation, RCS = right cathodal stimulation).

Supporting our hypothesis, the Bayesian directional analysis yielded evidence for the predicted one‐sided contrast pattern (BF = 31.086, *p*(*M*|data) = 0.969). As expected, and illustrated in Figure 2, PEP reactivity in the unfixed task was stronger following RCS (*M* = −7.44, SE = 1.19) compared to LCS (*M* = −6.13, SE = 1.24) and compared to both stimulation conditions during the fixed and easy task (left cathodal: *M* = −4.89, SE = 1.18; right cathodal: *M* = −5.03, SE = 0.90).

#### 3.3.2 SBP, HR, DBP Reactivity

The theory‐based a priori contrast for SBP, HR and DBP reactivity revealed low support for the models (BF = 1.456, *p*(*M*|data) = 0.593).^2^

Cardiovascular reactivity means and standard errors are presented in Table 2.

**TABLE 2:** Cell means and standard errors (in parentheses) of cardiovascular reactivity scores.

	LCS easy	RCS easy	LCS unfixed	RCS unfixed
	*M* (SE)	*M* (SE)	*M* (SE)	*M* (SE)
SBP (in mmHg)	7.45 (1.30)	5.46 (1.53)	7.50 (1.27)	6.40 (1.54)
HR (beats/min)	3.52 (1.02)	1.73 (1.00)	3.26 (1.07)	2.54 (1.06)
DBP (mmHg)	5.04 (1.12)	4.45 (0.90)	3.84 (1.14)	4.95 (1.25)

*Note:* *N* = 40 for SBP and DBP.

Abbreviations: DBP = diastolic blood pressure, HR = heart rate, LCS = left cathodal stimulation, RCS = right cathodal stimulation, SBP = systolic blood pressure.

### 3.4 Task Performance

Bayesian ANOVA on response accuracy favored the model that includes the main effects of stimulation and task plus their interaction (BF > 100, *p*(*M*|data) = 0.462). Analysis of specific effects showed low evidence for stimulation (BF_incl_ = 2.29), strong evidence for task (BF_incl_ > 100) with higher response accuracy in the fixed and easy task (*M* = 99.16; SE = 0.34) compared to the unfixed task (*M* = 91.53; SE = 0.93) and moderate evidence for stimulation × task interaction (BF_incl_ = 3.61). Response accuracy tends to be lower following RCS in the unfixed task (*M* = 89.58; SE = 1.34), compared to all other conditions (LCS unfixed: *M* = 93.48, SE = 1.16; LCS fixed and easy: *M* = 99.37, SE = 0.34; RCS fixed and easy: *M* = 98.95, SE = 0.6).

Bayesian ANOVAs for reaction times showed that the best model supports only the main effect of task (BF > 100, *p*(*M*|data) = 0.714). Reaction times were higher in the fixed and easy task (*M* = 769.94; SE = 36.03) compared to the unfixed task (*M* = 539.16; SE = 11.65). Models including stimulation and stimulation × task interaction revealed low Bayes factors and posterior model probabilities (BFs < 0.283, *p*(*M*|data) < 0.202). This provides little evidence that the stimulation or its interaction with task affected reaction times.

### 3.5 Funnel Debriefing

No participant correctly guessed the purpose of the experiment during the funnel debriefing. Furthermore, no participant reported pain associated with the experimental HD‐tDCS manipulation in any stimulation condition.

## 4 Discussion

To our knowledge, this is the first study to demonstrate that task context moderates the effects of HD‐tDCS on mental effort. RCS led to a higher effort‐related cardiac response when individuals worked on an unfixed task demand compared to LCS in the same task context and both stimulation conditions when participants worked on a fixed and easy task, as supported by Bayesian contrast. We attribute these results to RCS enhancing left FHA, which increases approach motivation and leads to heightened SR (e.g., Coan and Allen 2003; see Harmon‐Jones and Gable 2018; Kelley et al. 2017). In turn, this elevated SR likely causes individuals to place greater importance on achieving success when incentives are present, determining high effort. This is because MIT posits that success importance becomes the primary determinant of effort when task demand is unfixed (Brehm and Self 1989; see Gendolla et al. 2019; Richter et al. 2016 for recent reviews). However, when task demand is fixed and easy, MIT posits that success importance should have no effect on effort, leaving no room for observable neuromodulation effects on effort.

These results align with prior approach‐avoidance research (Fecteau et al. 2007; Framorando et al. 2025; Knoch, Gianotti, et al. 2006; Knoch, Pascual‐Leone, et al. 2006; Ohmann et al. 2018). Knoch, Gianotti, et al. (2006) showed that inhibitory TMS to the right dlPFC (left FHA) induced participants to prefer riskier decisions when associated with higher rewards compared to safer decisions but associated with lower rewards. Similarly, Ohmann et al. (2018) reported that left anodal stimulation (LAS) over the dlPFC (left FHA) increased participants' tendency to select more difficult trials associated with larger rewards over easier trials associated with lower rewards. Most relevant, similar effects had been observed in one of our previous lab's studies on effort‐related cardiac responses (Framorando et al. 2025). Specifically, participants worked on an unclear task demand and were informed that they could earn a moderate monetary reward in case of task success. Before completing the task, participants received either left cathodal, right cathodal or sham HD‐tDCS over dlPFC. Results showed that, for women only, RCS over the dlPFC led to higher effort during the mental concentration task compared to LCS and sham stimulation conditions. We attributed this result to RCS enhancing left FHA and increasing approach motivation. In turn, this likely led to higher SR, which increased success importance, resulting in higher effort (e.g., Coan and Allen 2003; see Harmon‐Jones and Gable 2018; Kelley et al. 2017). Taken together, these findings suggest that neuromodulation promoting LHA increases approach motivation and SR, determining higher effort to get rewards when success importance exerts a visible influence on effort.

Importantly, the present findings speak against a strictly autonomic account of tDCS effects on sympathetic activity (Carnevali et al. 2020; Sesa‐Ashton et al. 2022). Although earlier work showed that anodal dlPFC stimulation can alter autonomic indices such as heart rate and heart‐rate variability (Carnevali et al. 2020), a general sympathetic shift following stimulation cannot explain the current pattern. If HD‐tDCS merely triggered automatic autonomic activation, effort should have risen after both left and right cathodal stimulation across both task contexts (fixed and easy; unfixed). Instead, effort increased only following right cathodal stimulation when task demand was unfixed, whereas it stayed low following both stimulations in the fixed and easy task. This supports the idea that neuromodulation affected effort by changing the importance of success—consistent with MIT—rather than by eliciting a general sympathetic response.

In our Bayesian statistical analyses, participants showed increased effort‐related cardiovascular activity after RCS compared to LCS in the unfixed task and both stimulation conditions in the fixed and easy task, as revealed by PEP reactivity. However, our brain stimulation manipulation did not yield support for an effect on SBP, HR, or DBP reactivity. This finding aligns with the fact that PEP is the most sensitive measure of beta‐adrenergic sympathetic impact on the heart (Kelsey 2012; Richter et al. 2008; Wright 1996). SBP and, to an even greater extent, DBP are also affected by peripheral vascular resistance, rendering them less reliable effort indices than PEP. Also, HR is influenced by both sympathetic and parasympathetic activity. Therefore, effort effects on SBP and HR are less likely, and DBP effects are improbable. Importantly, in this study, PEP reactivity was not accompanied by simultaneous decreases in blood pressure or HR. Accordingly, the observed PEP responses can only be attributed to beta‐adrenergic sympathetic nervous system impact rather than cardiac preload or vascular afterload effects (see Sherwood et al. 1990).

Regarding performance, Bayesian analyses uncovered a moderate stimulation × task interaction on response accuracy. Accuracy decreased after RCS relative to LCS in the unfixed task and was comparably high under both stimulations when the task was fixed and easy. A strategy‐based explanation appears likely: in the unfixed task, participants earned the full monetary reward (USD 0.36) only when responses were both correct and fast, whereas correct but slower answers yielded just a third of that amount (USD 0.12). The heightened success importance presumed under RCS may therefore have prompted participants to favor speed, sacrificing accuracy to maximize payoffs. Correspondingly, mean reaction times tended to be lower following RCS in the unfixed task compared to the three other conditions, although Bayesian tests yielded no evidence for a stimulation × task effect on reaction times. Overall, these results reinforce the idea that stronger effort—indexed here by cardiovascular reactivity—does not automatically yield better performance, a divergence also noted in previous effort research (Framorando and Gendolla 2018, 2019a, 2019b; Gendolla and Silvestrini 2010; Framorando et al. 2021; Lasauskaite Schüpbach et al. 2014; Wang et al. 2023).

Importantly, recent studies suggest that tDCS effects, particularly over the dlPFC, may differ by sex (e.g., Gao et al. 2018; Lapenta et al. 2012; Palmisano et al. 2021; Weller et al. 2023; Yang et al. 2018). For instance, Gao et al. (2018) found that right anodal/left cathodal tDCS reduced deceptive behavior in women but had no effect on men, while Weller et al. (2023) reported greater cognitive performance gains in women than in men during tDCS over the dlPFC. Notably, Framorando et al. (2025) also reported a sex‐based interaction in tDCS effects on effort, where the expected tDCS effect was found only in women, while men showed a reversed pattern. Since our sample included only women, these results cannot be generalized to men, asking for replication in a mixed‐sex sample.

## 5 Conclusion

In summary, these findings provide the first evidence that—in reward‐based tasks—neuromodulation's effect on effort is context‐dependent. Right cathodal stimulation over the dlPFC increased effort‐related cardiovascular response compared to left cathodal stimulation within a self‐paced task where each correct trial makes participants earn money. In contrast, when the reward could be earned by merely maintaining at least 50% accuracy, cardiovascular effort remained low following both stimulation conditions. These findings suggest that neuromodulation affects effort by inducing changes in frontal hemispheric asymmetry, modifying the importance of earning monetary rewards rather than via automatic sympathetic activation—a global autonomic response that would increase cardiac reactivity regardless of task context.

## Author Contributions

**David Framorando:** conceptualization, investigation, funding acquisition, writing – original draft, methodology, writing – review and editing, project administration, data curation, supervision, formal analysis, software. **Sarah Delobel:** methodology, writing – review and editing, data curation. **Andrea Razzetto:** writing – review and editing, methodology, data curation.

## Conflicts of Interest

The authors declare no conflicts of interest.

## Data Availability

The data and data coding for the reported studies are available on the server from the GenevaMotivationLab: https://amb5.genevamotivationlab.ch/.

## Endnotes

^1^
Because EEG asymmetry has predominantly been investigated in right‐handed female participants (e.g., Harmon‐Jones and Allen 1997; Tomarken et al. 1992) and because neuromodulation effects on effort have been recently reported specifically in this population (Framorando et al. 2025), the present study recruited only right‐handed female participants. Importantly, the initial preregistration (https://aspredicted.org/hwbd‐67x4.pdf) did not restrict enrollment to female participants but instead targeted an equal sex split (50% male, 50% female), aiming for a total sample size of 100—twice the 51 female participants recruited in this study.

^2^
For SBP and DBP scores, one participant was excluded due to data loss, in addition to those initially excluded.
